# Use of a Cholera Rapid Diagnostic Test during a Mass Vaccination Campaign in Response to an Epidemic in Guinea, 2012

**DOI:** 10.1371/journal.pntd.0002366

**Published:** 2013-08-15

**Authors:** Isabel Martinez-Pino, Francisco J. Luquero, Kéïta Sakoba, Souleymane Sylla, Melatwork Haile, Rebecca F. Grais, Iza Ciglenecki, Marie-Laure Quilici, Anne-Laure Page

**Affiliations:** 1 European Programme for Intervention Epidemiology Training (EPIET), European Centre for Disease Prevention and Control (ECDC), Stockholm, Sweden; 2 Epicentre, Paris, France; 3 Ministère de la Santé et de l'Hygiène Publique, Conakry, Guinea; 4 Direction Préfectorale de la Santé, Forécariah Préfecture, Guinea; 5 Médecins Sans Frontières, Operational Center Geneva (MSF-OCG), Conakry, Guinea; 6 Médecins Sans Frontières, Operational Center Geneva (MSF-OCG), Geneva, Switzerland; 7 Enteric Bacterial Pathogens Unit, National Reference Centre for Vibrios and Cholera, Institut Pasteur, Paris, France; Massachusetts General Hospital, United States of America

## Abstract

**Background:**

During the 2012 cholera outbreak in the Republic of Guinea, the Ministry of Health, supported by Médecins Sans Frontières - Operational Center Geneva, used the oral cholera vaccine Shanchol as a part of the emergency response. The rapid diagnostic test (RDT) Crystal VC, widely used during outbreaks, detects lipopolysaccharide antigens of *Vibrio cholerae* O1 and O139, both included in Shanchol. In the context of reactive use of a whole-cell cholera vaccine in a region where cholera cases have been reported, it is essential to know what proportion of vaccinated individuals would be reactive to the RDT and for how long after vaccination.

**Methodology/Principal Findings:**

A total of 108 vaccinated individuals, selected systematically among all persons older than one year, were included at vaccination sites and 106 were included in the analysis. Stools samples of this cohort of vaccinated participants were collected and tested with the RDT every day until the test was negative for two consecutive visits or for a maximum of 7 days. A total of 94.3% of cholera vaccine recipients had a positive test after vaccination; all except one of these positive results were reactive only with the O139 antigen. The mean time to become negative in those with an initial positive result after vaccination was 3.8 days, standard deviation 1.1 days.

**Conclusions/Significance:**

The RDT Crystal VC becomes positive in persons recently vaccinated against cholera, although almost exclusively to the O139 antigen. This reactivity largely disappeared within five days after vaccination. These results suggest that the test can be used normally as soon as 24 hours after vaccination in a context of O1 epidemics, which represent the vast majority of cases, and after a period of five days in areas where *V. cholerae* O139 is present. The reason why only O139 test line became positive remains to be investigated.

## Author Summary

The rapid diagnostic test (RDT) Crystal VC detects lipopolysaccharide antigens from V. *cholerae* O1 and O139 in stool samples, which are also present in the oral cholera vaccine Shanchol. It is important to take into consideration the possibility of a positive result to the RDT due to vaccination and not to cholera in recently vaccinated individuals. During a large mass cholera vaccination campaign in Kabak (Guinea) in 2012, we conducted a study to estimate the proportion of positive results to the RDT in recipients of the oral cholera vaccine at different time points after vaccination. The results of this study show that ingestion of the cholera vaccine led to a positive RDT, although almost exclusively to the O139 antigen, in the majority of vaccinated people. From the fifth day after vaccination, only a small minority of vaccinated individuals remained positive for the RDT and none of the specimens tested the seventh day of follow-up were positive. Our findings provide the first data on the use of the RDT Crystal VC in vaccinated people. This test should be used carefully during the first week after reactive mass oral cholera vaccination campaigns in areas where *V. cholerae* O139 is present.

## Introduction

Cholera is an acute diarrhoeal infection caused by ingestion of the bacterium *Vibrio cholerae*. Two serogroups– O1 and O139 – are responsible for cholera epidemics. While *V. cholerae* O1 causes the majority of outbreaks over the world, O139 – first identified in Bangladesh in 1992 – is confined to South-East Asia [1], where its incidence has declined over the years [2]. Globally, O139 accounts for a small minority of cholera cases [3], and local transmission has never been reported in Africa or America. Rapid identification of initial cases of cholera in the early phase of an epidemic is critical for implementation of a timely public health response [4] to control the spread and duration of the outbreak. Currently, cholera diagnosis relies on the microbiological identification of the pathogen by stool culture, which remains the gold standard to confirm the diagnosis [5]. However, this procedure requires laboratory infrastructure, adequate transport procedures and trained staff [5]. As rapid diagnostic tests (RDT) require less time, a minimum laboratory infrastructure and basic technical skills, they are used to confirm cholera outbreaks in places where high laboratory standards are difficult to obtain [6].

In 2003, the Institut Pasteur developed a cholera RDT based on the qualitative detection of lipopolysaccharide (LPS) antigen of both *Vibrio cholerae* O1 and O139 serogroups from stool specimens. This test uses one-step, vertical-flow immunochromatography principle and monoclonal antibodies against the core and O-specific polysaccharides of each serogroup for capture and detection of antigens [7], [8]. The O1 specific antigenic determinant is common to Ogawa and Inaba serotypes [8], [9] and the one for O139 is common to both O139 capsular polysaccharide and LPS. This cross-reactivity between O139 LPS and capsular polysaccharide explains that antibodies react with both encapsulated and non-encapsulated *V. cholerae* O139 strains [10]. The RDT is produced by Span Diagnostics (Surat, India) under the trade name Crystal VC [5]. Several evaluations have shown good sensitivity, ranging from 92% to 100% [7], [11]–[12]. In contrast, the specificity was lower and most evaluations in field conditions have shown specificities from 71% to 77% when compared with culture as the gold standard [4], [11]–[13]. Nevertheless, the use of culture as gold standard may underestimate specificity, and re-analysis of the data using statistical methods for evaluation with an imperfect gold standard showed that the specificity could be around 85% [14]. After these evaluations, the manufacturer SPAN changed the test presentation (order of the lines and addition of a dilution buffer), but the test in this new version has not been formally evaluated. This test is widely used for epidemiological purposes during outbreaks.

In 2012, the Republic of Guinea faced an O1 cholera epidemic, with the first cases notified in the prefecture of Forécariah in February. In light of the ongoing cholera epidemic and the 2009 World Health Organization (WHO) recommendations calling for the consideration of oral cholera vaccines as a part of the epidemic response [15], the Ministry of Health and Public Hygiene (MHPH) of Guinea supported by Médecins Sans Frontières – Operational Center Geneva (MSF-OCG), implemented a vaccination campaign in the prefectures of Boffa and Forécariah. The vaccine Shanchol (Shantha Biotechnics, India), prequalified by the WHO, contains killed bacteria *V. cholerae* O1 and O139 and, given in two doses 14 days apart, provides nearly 70% protection for at least 2 years after vaccination [16]. A total of 7,531 cases including 138 deaths (case fatality ratio of 1.8%) were reported to the MHPH of Guinea between the beginning of the epidemic and its end, which was declared on 6 February 2013, after six consecutive weeks without any new case notification [17].

Given that the RDT Crystal VC detects the LPS antigens of *V. cholerae* O1 and O139 in feces, which are also contained in the oral vaccine Shanchol, we hypothesized that the stools of vaccinated individuals could become positive by the rapid test due to the vaccine only, in the absence of viable bacteria. In a reactive campaign during an outbreak, positive test results due to the vaccine could interfere with the use of the tests in suspected cholera cases. The aim of this study was to estimate the proportion of positive results of the test Crystal VC in recipients of the cholera vaccine Shanchol at different time points after vaccination and the mean time to become negative (in those with an initial positive result for O1 or O139) after vaccination.

## Methods

### Ethics statement

The study protocol was approved by the Ethical Review Board (ERB) of Guinea and the MSF ERB. Written informed consent was obtained from adults or from the guardians of participants less than 18 years of age. Privacy and confidentiality in the data collected from the participants were ensured both during and after the conduct of the study.

### Setting, population and study design

The study took place in Kabak (Forécariah Prefecture, Guinea) during the second round of the mass vaccination campaign carried out by the MHPH/MSF in June 2012. The study population corresponded to the population targeted by the vaccination campaign (all residents of Kabak aged one year and above). Individuals were included if they were vaccinated and accepted to participate. They were excluded if they had watery diarrhea on inclusion (to exclude potential cholera cases) and/or a high probability of not being present for all the follow-up visits. The cohort of vaccinated participants meeting study criteria was followed-up prospectively.

We estimated that 96 individuals were needed to achieve a minimum precision of 10% around a proportion of 50% of positive RDT, as there were no data on the prevalence of positive tests in the vaccinated population. We increased the sample size to 106 to account for an expected 10% of loss to follow-up. A systematic sampling method (one every 10 individual) was used in every vaccination site.

### Recruitment and follow-up procedures

Participants were recruited in 4 of the 31 vaccination sites, selected arbitrarily, as vaccination sites were not thought to have any influence on the study outcomes. Demographic information was collected at inclusion through a face-to-face interview (mainly in Soussou, the local language) and information on stool production and basic clinical symptoms during follow-up visits using an individual standardized case report form (CRF). Participants were asked to collect stool in a pot provided by the study team. Participants' homes were visited daily to collect stool specimens, complete a follow-up form and to provide them with a new pot for the next stool. We transported the stools to the laboratory and tested them with the RDT. Laboratory technicians completed the information with the RDT results. Follow-up was considered finalized when 2 consecutive negative RDT results were obtained or after 7 days.

### Field use of the rapid diagnostic test

The stool samples were tested with the RDT at Kabak Health Center following the manufacturer's instructions by a laboratory technician trained to the use of the test. Crystal VC tests used were manufactured in 2011 and 2012 by Span Diagnostics Ltd., India (catalogue reference number 161C101-10). A small portion of stool was mixed with a buffer and 200 µL (4 drops) of the mix was placed in a test tube. The dipstick test was left in the tube for 20 minutes before reading. If only the control line appeared, the test was negative. If 2 or 3 lines appeared, the test was positive for either *V. cholerae* O139, O1, or both. If the control line was absent, the test was considered invalid and repeated once.

### Laboratory control of the rapid diagnostic test

Ten by ten dilutions of the Shanchol vaccine were prepared using the dilution buffer provided in the RDT kit. Undiluted and diluted vaccine solutions up to a 10^9^-fold dilution were tested with the RDT following the manufacturer's recommendations.

A bacterial suspension adjusted to an optical density at 600 nm (OD_600 nm_) of 0.8 was prepared in the dilution buffer provided in the RDT kit from an overnight culture of *V. cholerae* O1 and O139 strains. Such an OD value was previously estimated to correspond to 2×10^8^ *V. cholerae*/mL by colony counting of 10­fold serial dilutions spread on agar plates and incubated over night at 37°C. This initial solution was used to prepare solutions at 2×10^7^ and 2×10^6^ bacteria/mL using the dilution buffer provided in the kit, undiluted and diluted solutions were tested with the RDT following the manufacturer's recommendations.

### Data analysis

Qualitative variables were described through their frequency and percentages. Continuous variables were described through their mean, median, standard deviation (SD) and percentiles (P_25_ and P_75_). We calculated the proportion of positive results for O1 or O139 for each day of follow-up including in the numerator the number of positive results and in the denominator the sum of the total number of tests performed and the number of cases for whom follow-up was stopped after obtaining two consecutive negative results. Missing data (absent or no stool sample) were excluded from this calculation. The 95% exact confidence intervals (95%CI) of the proportion estimate were calculated. To estimate the mean time to obtain a negative RDT result after vaccination (time to become negative) we counted the number of days needed to obtain a first negative result in the group of people who obtained previously a positive result for O1 or O139 after vaccination. Statistically significant differences by gender and age were assessed with a linear regression model. A p value<0.05 was considered significant.

Data were entered in an EpiData version 3.1 database (EpiData, Odense, Denmark) and analyzed using Stata version 11 (StataCorp, College Station, Texas, USA).

## Results

### Recruitment and follow-up

A total of 108 individuals were recruited during 2 days in 4 vaccination sites. Two individuals were excluded from the analysis (one was absent during all follow-up visits and for the other, follow-up was stopped accidentally by the study team).

**Figure 1:**
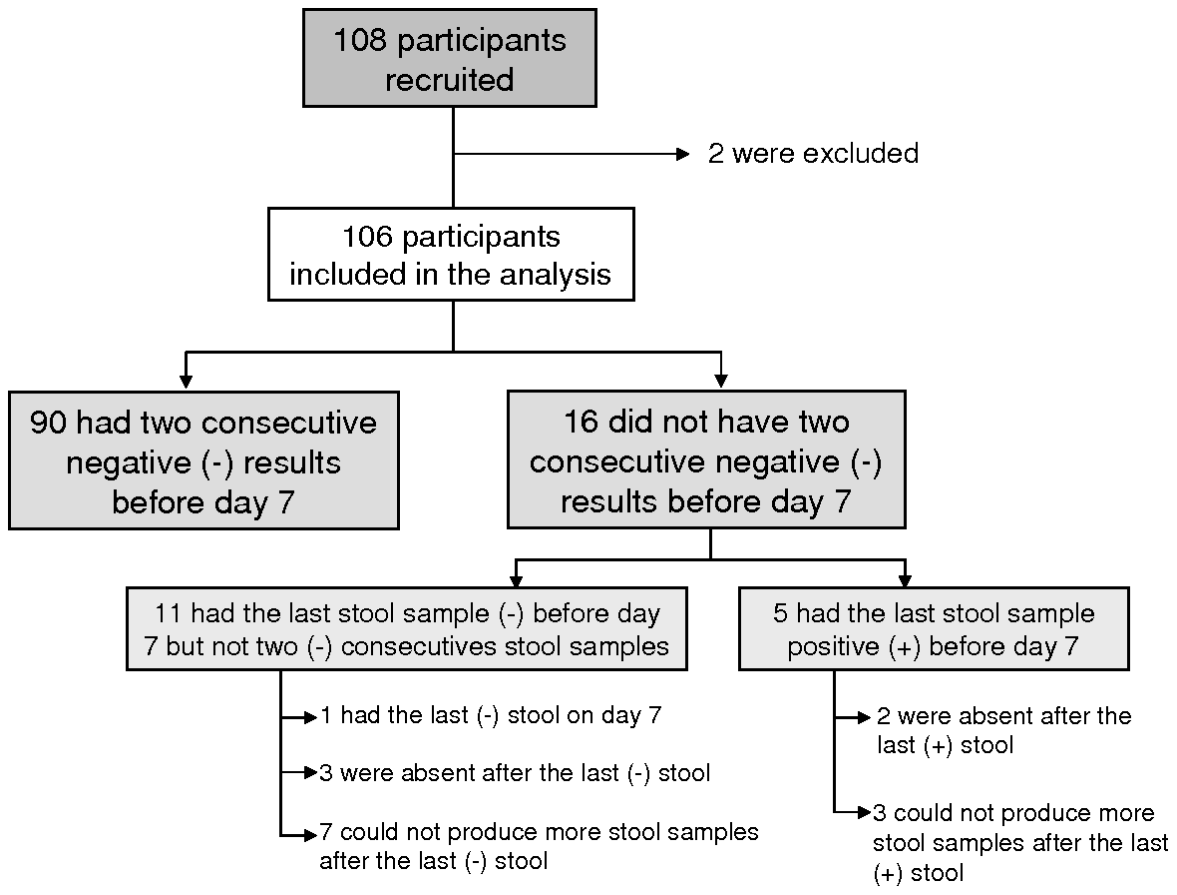
Study participants, exclusions and follow-up results, Kabak, 2012.

Follow-up of the remaining 106 participants is described in Figure 1. Participants were followed for a median time of 5 days (minimum of 2 and 7 as maximum). Almost half of them (49.1%) were followed for 4 (23.6%) or 5 days (26.4%).

### Participant's characteristics, symptoms and delay in stool collection and testing

Among the 106 participants, 79.2% (84) were females and the median age was 25 years (P_25_-P_75_ = 2–80). The majority of participants were older than 15 (84.8%) and the proportion of children under five was 5.7%.

In total, 18 participants declared having diarrhea during follow-up, and two reported vomiting. Other symptoms such as constipation, stomachache or headache were declared by 37 participants.

The average delay was 3.9 hours (SD = 4.4) between stool production and collection and 6.6 hours (SD = 5.9) between stool collection and performance of the RDT (including collection and transport of samples to the laboratory) by the laboratory technicians. As a result, there was an average delay of 10.5 hours (SD = 6.6) between stool production and performance of the RDT.

### Proportion of positive tests after vaccination

Of the 106 participants, 100 (94.3%) became positive with the O139 line after vaccination and 6 never had a positive result. On the first day of follow-up (day 1) 71.1% were positive. On day 3, almost half of the tests remained positive (49.5%) and on day 5 and 6 this percentage decreased below 3% (Table 1).

**Table 1:** Rapid diagnostic test results in vaccinated participants by day of follow-up, Kabak, 2012.

	Day 1	Day 2	Day 3	Day 4	Day 5	Day 6	Day7
**A. Tests performed**	97	97	90	76	46	23	6
a.1. Positive result (+)	69	80	47	20	2	1	0
a.2. Negative result (−)	28	17	43	56	44	22	6
**B. Follow-up stopped after 2(−)**	0	0	5	17	42	67	85
**C. Absent**	1	0	0	1	1	4	5
**D. No sample available**	8	9	11	12	17	12	10
**Total** 1	106	106	106	106	106	106	106
**Proportion** 2 **of positives (%)**	71.1	82.5	49.5	21.5	2.3	1.1	0.0
**95%CI of the proportion**	61.5–79.9	73.4–89.4	39.1–59.9	13.7–31.2	0.3–8.1	0.0–6.0	0.0–4.0^3^

1 The total is the sum of A+B+C+D.

2 The proportion is the result of the formula (a.1/(A+B))*100.

3 97.5% Confidence Interval, one-sided.

Only one participant became positive with the O1 line (together with the O139 line) on the first day of monitoring, and both lines became negative subsequently.

### Time to become negative

Of the 100 participants with at least one positive result, five could not be tested on day 7 as they were absent or did not produce stools, although they had a positive result with their last specimen collected (Figure 1). Among these 5 participants, 3 had their last positive stool on day 3, 1 on day 4 and 1 on day 5. For the remaining 95 cases with O139 positive tests, we calculated the time to become negative after vaccination.

For all participants, the mean time to become negative after vaccination was 3.8 days (SD = 1.1) and the median time was 4 days (P_25_-P_75_ = 3–5). For males, the mean time to become negative after vaccination was 4.3 days (SD = 1.4) and 3.6 (SD = 1) for females (p = 0.03), with a median of 4 days for both males and females. A linear regression model showed that a longer time to become negative was associated to an older age (p = 0.002) and to male sex (p = 0.012) (Table 2).

**Table 2:** Linear regression model of time to become negative by age and sex, Kabak, 2012.

	Coefficient	95% Confidence Interval	p
**Age** 1	0.020	(0.008–0.032)	0.002
**Sex** 2	0.669	(0.153–1.186)	0.012

1 The coefficient shows the increase in days in the time to become negative per year of age.

2 The coefficient shows the increase in days in the time to become negative for males compared to females.

### Laboratory testing of the rapid diagnostic test

The Crystal VC RDT gave positive results for both O1 and O139 when the strip was inserted directly into the vaccine solution prior to ingestion, and remained positive up to 10^4^-fold dilutions of the vaccine. At a 10^5^-fold dilution, only the O139 line remained positive and none of them were positive at higher dilutions (Table 3).

**Table 3:** Rapid diagnostic test results performed in vaccine and bacterial suspension dilutions, Pasteur Institute, 2012.

	Control line	Line T1 O139	Line T2 O1
**Vaccine dilutions**			
Tube 1 (10-fold dilution)	+++	+++	+++
Tube 2 (10^2^-fold dilution)	+++	+++	+++
Tube 3 (10^3^-fold dilution)	+++	+++	++
Tube 4 (10^4^-fold dilution)	+++	++	+
Tube 5 (10^5^-fold dilution)	+++	+	−
Tube 6 (10^6^-fold dilution)	+++	−	−
Tube 7 (10^7^-fold dilution)	+++	−	−
Tube 8 (10^8^-fold dilution)	+++	−	−
Tube 9 (10^9^-fold dilution)	+++	−	−
**O1 and O139 strains dilutions**	+++		
O1 - Tube 1 (2×10^8^bacteria/mL)	+++	−	+++
O1 - Tube 2 (2×10^7^ bacteria/mL)	+++	−	++
O1 - Tube 3 (2×10^6^ bacteria/mL)	+++	−	−
O139 - Tube 1 (2×10^8^ bacteria/mL)	+++	+++	−
O139 - Tube 2 (2×10^7^ bacteria/mL)	+++	+++	−
O139 - Tube 3 (2×10^6^ bacteria/mL)	+++	++	−

Intensity of the positive line: (+) very weak positive; (++) weak positive; (+++) positive.

Negative result: (−).

The RDT gave a positive signal with the O1 test line at bacterial concentration of 2×10^8^ and 2×10^7^, but was negative at 2×10^6^ bacteria/mL, while all dilutions of *V. cholerae* O139 culture tested down to 2×10^6^ bacteria/mL were positive for the O139 line (Table 3).

## Discussion

To our knowledge, this is the first study showing that healthy individuals vaccinated with the oral vaccine Shanchol become positive with the cholera rapid test Crystal VC in the first days following vaccination. The proportion of vaccinated individuals positive for the Crystal VC test after vaccination was high (94.3%) for the O139 component of the test, but low with the O1 component. This proportion of O139-positive tests decreased rapidly to half on the third day after vaccination and to one-fifth on the fourth day of follow-up. The median duration required to have a negative result for those cases presenting a previous positive test was 4 days.

Almost all positive tests (except for one) were positive only for O139 line, despite the fact that the Shanchol vaccines contains the two strains *V. cholerae* O1 and O139, with a higher amount of O1 (1500 Elisa units of *V. cholerae* O1 LPS and 600 Elisa units of *V. cholerae* O139 LPS for a dose of 1.5 mL) [18]. This could be due to a higher sensitivity of the RDT for the O139, as suggested by the results of sensitivity against bacterial cultures showing that the O139 line was reactive with higher bacterial dilutions than the O1 line. Such results were already reported by Nato et al. [7] when evaluating the initial version of the RDT, but are in contradiction with those observed by Mukherjee et al. [13] with the first version of the Crystal VC test, which was reactive at 10^6^ bacteria/mL for *V. cholerae* O1 and 10^7^ bacterial/mL for *V. cholerae* O139. These differences of analytical sensitivity between the different versions of the RDT emphasize the need for a proper diagnostic performance evaluation of each new version of the test.

Including pre-vaccination stool status of our study population as well as unvaccinated participants could have provided useful information on the magnitude of potential false positive reactions due to factors unrelated to vaccination, i.e. non-specific reactions, which could have been expected considering the reported moderate specificity of the test [4], [11]–[13], or positivity due to asymptomatic carriers. The sharp increase and subsequent decrease in the proportion of O139 positive tests after vaccination are not in favour of such assumptions and suggest that the positive results were due to the vaccine alone. Of the 75 tests done after day 5, only three (4%) were positive for O139, and overall only one test was positive for O1 which is lower than the number of false positives that could be expected based on the test specificity. However, it should be noted that this study was conducted in people without cholera symptoms while the previous evaluations were conducted in suspected cholera cases.

There are several limitations worth noting. First, women and adults were overrepresented in our study sample. Although women were more vaccinated than men were during the vaccination campaign carried out in Kabak, the proportion of women in our study (79.3%) was clearly higher than the vaccinated population (59.5%) [19]. This is likely due to the fact that the majority of men presented early at the vaccination site and were more likely to be excluded given their potential absence for work during the follow-up period. However, although there was a small difference in the mean time to become negative between men and women (4.3 days vs. 3.6), the median was the same for both sexes (4 days) thereby not likely affecting the results presented here. The median age in the study was 25 years compared to 15 for the vaccinated population [19]. Considering that the time to become negative was longer for the older participants, it is likely that we slightly overestimated the time to become negative. Nonetheless, the differences by age were small in magnitude (0.2 days per 10 years of age) and they do not change the interpretation of the results neither our recommendations regarding the use of the cholera RDT in vaccinated areas. Second, we could not conclude on five cases who had a positive result with their last specimen collected, and for whom further samples could not be collected because they were absent or unable to produce stool samples. When designing the study, we decided to limit the follow-up period to 7 days, based on the expected time for gastrointestinal transit of the killed bacteria. Although extending the follow-up of participants until they became negative for the rapid test would have been useful for concluding on these 5 individuals, we consider that this limit was reasonable in the absence of any other data. In addition, even if we consider that these five people were still positive at day 7, the percentage of positive tests would be still low (5.2%), lower than the expected for non-cholera cases considering the specificity of the test. Third, we did not perform culture to exclude participants with possible cholera or asymptomatic carriage of *V. cholerae*. Although initially planned in the protocol for participants with diarrhea or with a positive RDT at the end of follow-up (day 7), no culture was performed since symptoms were found unreliable and none of the specimens tested on the seventh day of follow-up were positive. Finally, specimens were tested on average ten hours after stool production and without the possibility of storage at 4°C due to the lack of electricity in Kabak. This delay seems reasonable given the difficulties to collect the samples immediately after production, although it is unclear the degree to which antigens degrade during this period, which could potentially affect the RDT results.

The results of the study confirm our hypothesis that the rapid test Crystal VC can become positive in persons recently vaccinated against cholera, although only with the O139 line, probably linked to its higher analytical sensitivity. However, tests become negative rapidly and five days after vaccination the proportion of positive tests among vaccinated is less than 3%. As the current global pandemic is almost exclusively caused by *Vibrio cholerae* O1, our results suggest that the current Crystal VC kit can be used normally as soon as 24 h after receiving Shanchol in a context of *V. cholerae* O1 epidemic, and after a period of five days in areas where *V. cholerae* O139 is present. Other cholera rapid diagnostic tests based on the LPS detection are available in the market [20] and could also become positive in recently vaccinated individuals. Thus, an evaluation of other tests or future versions of the Crystal VC test is recommended if they are to be used in the context of oral cholera vaccination campaigns. Finally, we strongly recommend that the diagnostic performances of the current modified version of the Crystal VC test be evaluated with respect to the different sensitivities of the O1 and O139 lines.

## Supporting Information

Checklist S1 **STROBE checklist.**
(PDF)
